# Using Cu–Zn–Sn–O Precursor to Optimize CZTSSe Thin Films Fabricated by Se Doping With CZTS Thin Films

**DOI:** 10.3389/fchem.2021.621549

**Published:** 2021-04-16

**Authors:** Qian Li, Jinpeng Hu, Yaru Cui, Juan Wang, Yu Hao, Tong Shen, Lizhen Duan

**Affiliations:** Laboratory of Vacuum Smelting, School of Metallurgical Engineering, Xi’an University of Architecture and Technology, Xi’an, China

**Keywords:** Cu–Zn–Sn–O, precursor, nanoink, Cu_2_ZnSn(S,Se)_4_ thin film, solar cell

## Abstract

The copper–zinc–tin oxide (CZTO) precursor was synthesized to avoid sudden volume expansion from CZTO precursor to Cu_2_ZnSnS_4_ (CZTS) thin films and smooth CZTSSe thin-film surfaces without pinholes. The CZTO precursor was prepared by coprecipitation and ball milling to form nanoink of CZTO. Based on the CZTO precursor, the CZTS thin film was fabricated and then selenized to make pinhole-free and flat Cu_2_ZnSn(S,Se)_4_(CZTSSe) thin films. The results show that the CZTO precursor greatly contributed to elevating the homologous surface characteristics and crystallinity of CZTSSe thin films by controlling selenium temperature, selenium time, and selenium source temperature. Finally, the conversion efficiency of the CZTSSe thin-film solar cell fabricated from the CZTO precursor was 4.11%, with an open-circuit voltage (Voc) of 623 mV, a short circuit current density (Jsc) of 16.02 mA cm^−2^, and a fill factor (FF) of 41.2%.

## Introduction

In recent years, with the emergence of environmental problems and resource depletion, more scholars have paid their attention to green and sustainable materials and devices (Ali et al., 2019; Sharma et al., 2019). Cu_2_ZnSnS_4_(CZTS) is nontoxicity with elements, low cost, pollution-free, excellent bandgap (1.4–1.5 eV), and absorption coefficient (10^4^/cm) (Wen et al., 2015; Kumar et al., 2018; Sun R. et al., 2019). Due to those advantages, CZTS thin-film material has attracted extensive attention. Up to now, CZTS thin films have been prepared by many methods such as sol–gel technique (Abdolahzadeh Ziabari et al., 2020), electrodeposition (Pawar et al., 2010), chemical bath deposition (Wang and Gong, 2011), and electrospinning technique (Ennaoui et al., 2009).

However, the direct generation of Cu_2_ZnSnS_4_(CZTS) using Cu–Zn–Sn(CZT) is always accompanied by the appearance of a secondary phase and defects, which make the morphology of thin film worse (Yoo et al., 2012). It was found that the preparation of CZTS with the copper–zinc–tin oxide (CZTO) precursor could effectively improve the morphology and smooth the surface of the thin film, and O atoms can be completely replaced by S atoms after the sulfidation procedure without residual oxide impurities (Ishino et al., 2013; Liu et al., 2018).

On the other hand, the semiconductor materials still present several defects such as large volume resistivity, defect density, and lower short circuit current (Jsc) causing poor device performance (Li et al., 2020). Tang prepared CZTS thin films by coprecipitation method, but the conversion efficiency was only 1.22% (Tang et al., 2013). Dong formed the CZTS absorption layer by simple sol–gel spin coating on Mo glass sheet, and the conversion efficiency of the solar cell was 2.25% (Dong et al., 2017). In order to prepare solar cells with high efficiency, Se doping is usually used to promote the growth of thin films (Chen et al., 2018). Chawla synthesized CZTSSe by high temperature arrested precipitation method (Chawla et al., 2018), with the increase of Se/S ratio, the degree of crystallization increases, and the calculated bandgap value was close to the optimal value of the solar photovoltaic conversion. Jin prepared CZTSSe thin films with CZTO as a precursor, the CZTO particles ensure the homogeneous distribution, and the conversion efficiency of 4.94% was achieved (Jin et al., 2016).

In this study, the CZTO precursor was introduced to reduce volume expansion of the thin film, inhibit the formation of the second phase, and also improve the property of thin-film solar cells. A smooth surface of a CZTSSe thin film without pinholes was obtained by annealing the CZTO precursor profiting for atoms’ entry into the precursor and improving crystal growth. The effects of controlling annealing temperature, holding time, and selenium source temperature on the microstructure and phases of CZTSSe thin films were studied providing a theoretical basis for the preparation of thin-film solar cells with low cost and high performance.

## Experimental

### Synthesis of CZTO Thin Films

In a typical procedure, copper sulfate, zinc sulfate, and stannic sulfate were added with surfactant-increasing and pH-regulating method; the obtained Cu–Zn–Sn hydroxide was gathered and purified by filtration using DI water and ethanol three times and then calcined at 550°C to produce the CZTO nanoparticles. The mixture containing a certain amount of ethanol and CZTO nanoparticles was ball milled to form a nanoink, immediately, spinning on the Mo substrate to obtain the CZTO precursor thin film.

### Sulfidation of CZTS Thin Films

The CZTO thin film and sulfur powder were separately placed on the downstream side and the upstream side of the tube furnace. The sample was sulfide at 580°C for 1 h under Ar atmosphere with a heating rate of 10°C/min. Finally, the CZTS thin film was generated.

### Selenization of CZTSSe Thin Films

In order to optimize the performance of the thin film, the exceeded selenium powder and CZTS thin film were separately placed on the left side and right side of the tube furnace in the process of Se doping. The sample was selenized at 450°C for 1 h under Ar atmosphere with a heating rate of 10°C/min to obtain the desired CZTSSe thin film. In comparison, the annealing temperatures were 400, 500, 550, and 580°C, respectively, starting at different times of 0.5, 1.5, and 2 h and different Se source temperatures of 350, 400, and 450°C, respectively.

### Preparation of CZTSSe Solar Cells

Solar cells with the structure Mo/CZTSSe/CdS/i-ZnO/ITO/Ag were fabricated. The N-type CdS layer was deposited on the CZTSSe layer by chemical bath method, and the i-ZnO and ITO windows layer were synthesized by RF magnetron sputtering and DC magnetron sputtering. Ag glue was pasted on top of the devices. Finally, the effective regions of the devices were 0.09 cm^−2^.

### Characterization

The characterization of the CZTSSe thin film from CZTO precursor prepared by different annealing parameters was implemented with several methods of measurement. The surface and cross-sectional morphologies of CZTO precursor and CZTSSe thin films were observed using field emission scanning electron microscopy (FESEM, NOVA450) at 20 kV. The elemental composition of the CZTSSe thin film was estimated using an X-ray energy spectrometer (EDS) attached to the FESEM and the thickness of the thin film was determined with a surface profiler (Veeco Dektak 150). The crystal structure of the CZTSSe thin film was analyzed by X-ray diffraction (XRD, Rigaku D/MAX-2000H) with CuKα radiation at 40 kV and 300 mA. To further confirm the crystallinity of the CZTSSe thin film, the phase was also performed using a Raman spectrometer (HR800-Horiba) with a laser excitation wavelength of 488 nm. The current–voltage (J-V) characteristics of CZTSSe solar cells were measured on an Oriel mode 94023 A measurement system.

## Results and Discussion

### Microstructure Characterization of CZTO Precursor Thin Films

**FIGURE 1:**
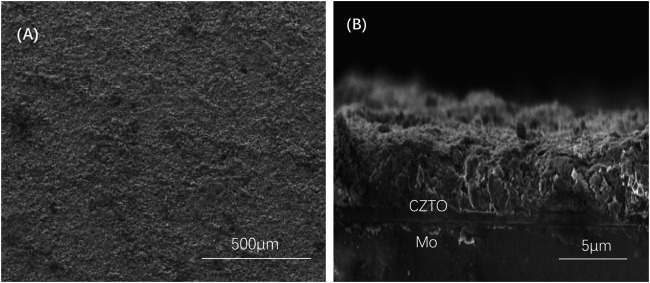
Surface SEM image **(A)** and cross-sectional SEM image **(B)** of CZTO precursor thin film.

Figure 1A shows the SEM image of the CZTO precursor thin film fabricated by spraying method with nanoink. The formed CZTO thin film by annealing owned good grain size and flat substrate. In the cross-sectional image of Figure 1B, the thickness of CZTO was approximately optimum, achieving perfect contact with Mo-based substrates without obvious void inside the membrane.

### Selenization Effect Under Different Selenization Temperatures

The ratio of CZTSSe elements Cu/(Zn + Sn) was 1.02, 0.90, 1.04, 0.98, and 0.86 for the selenization temperature at 400, 450, 500, 550, and 580°C, and the ratio of Zn/Sn was 1.02, 0.90, 0.92, 1.01, and 0.84 at 400, 450, 500, 550, and 580°C, respectively. The data provide information that the sample keeps Cu-poor and Zn-rich compositions under 550°C of selenization temperature. On the one hand, Cu-poor condition causes more vacancies to be created and the concentration of shallow acceptor increased; besides, Zn-rich condition could restrain self-compensation defects and further improve the photoelectric conversion efficiency of CZTSSe thin-film solar cells (Chen et al., 2013; Sun L. et al., 2019).

**FIGURE 2:**
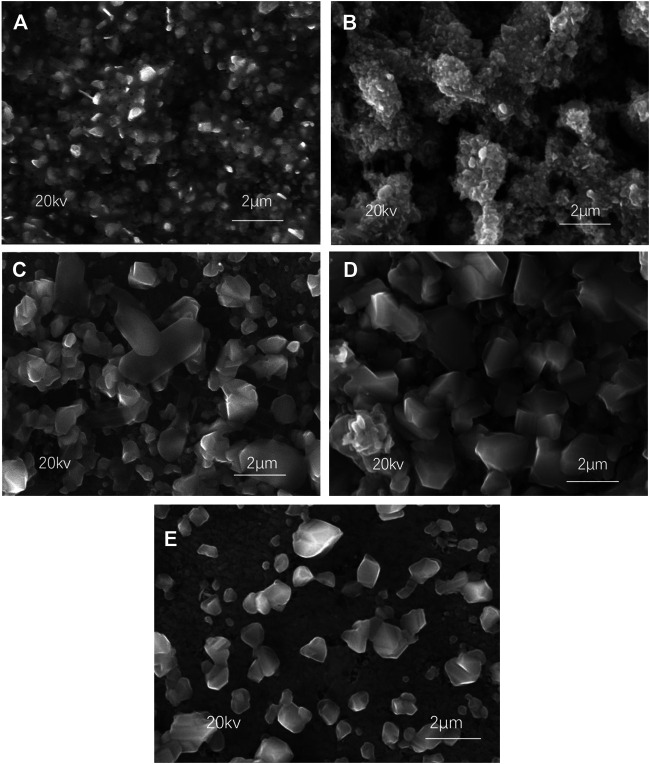
SEM images of CZTSSe thin films annealed at different temperatures: **(A)** 400°C, **(B)** 450°C, **(C)** 500°C, **(D)** 550°C, and **(E)** 580°C.

Figure 2 shows the SEM images of CZTSSe surfaces at different selenization temperatures. As shown in Figures 2A,B, both CZTSSe particles exhibited small-sized crystals, suggesting that low substrate temperature was insufficient to form large-sized crystals. After annealing at 500°C, the CZTSSe particles can be clearly identified but showed an uneven distribution as shown in Figure 2C. The particle agglomeration of CZTSSe thin films was significantly reduced and CZTSSe demonstrated uniform distribution with an approximate size of 60 nm in Figure 2D at 550°C, indicating that the morphology of the thin film was improved. Nevertheless, after continuous heating to 580°C, the thin film was uneven again along with the loose surface.

**FIGURE 3:**
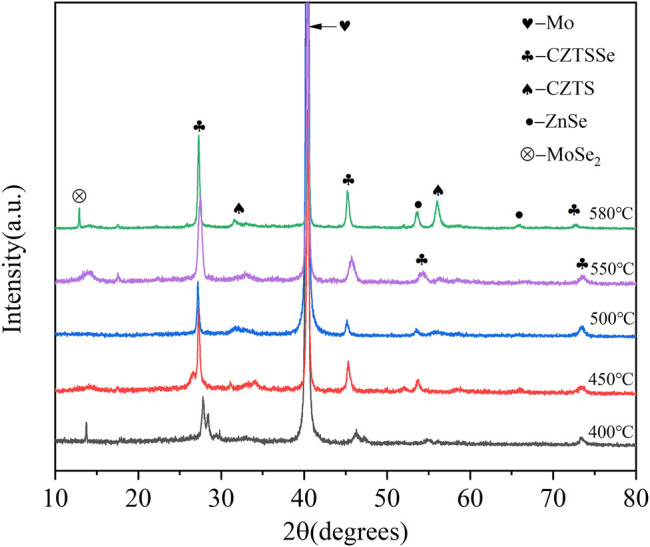
XRD patterns of CZTSSe thin films annealed at different temperatures 400, 450, 500, 550, and 580°C.

The XRD curves of CZTSSe thin films at different selenium temperatures are shown in Figure 3. Compared to the standard PDF cards (CZTS: JCPDS No. 26–0575 and CZTSe: JCPDS No. 52–0868), the three strong peaks in the figure can be completely matched, which proved the high crystallinity of CZTSSe (Ren et al., 2020; Sakul Bal et al., 2020). The diffraction peaks at 27.5°, 46.3°, and 54.1° were distinct and sharp at 400°C, and both peaks can be directed to the CZTS and CZTSe phase proving the presence of CZTSSe (Joshi et al., 2019). The diffraction peak had the smallest full width at half maximum when temperature arrived at 550°C, indicated of the best crystallinity. However, the secondary phases of MoSe_2_ and ZnSe appeared at 580°C, caused by the reaction of decomposition products of CZTS with Se and Mo substrate, resulting in a lower short circuit current. It was certificated that excessive temperature caused the formation of secondary phase resulting in material defect structure type and concentration hard to control.

**FIGURE 4:**
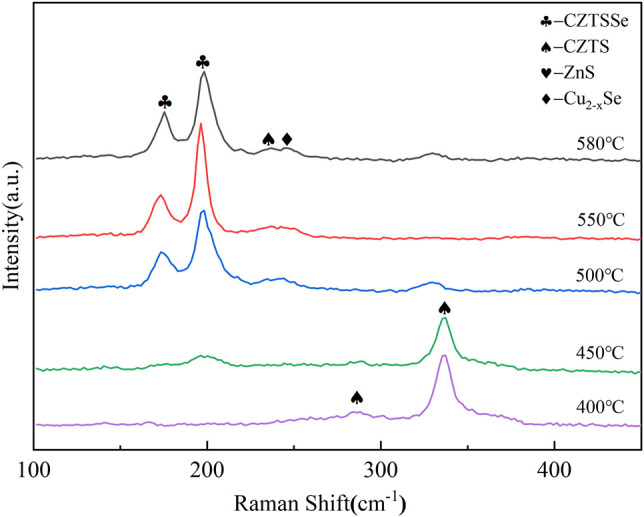
Raman spectra of CZTSSe thin films annealed at different temperatures 400, 450, 500, 550, and 580°C.

The Raman diagrams of CZTSSe thin films at different selenization temperatures are shown in Figure 4. The main peak of 336 cm^−1^ represented the generation of CZTS at 400°C (Khare et al., 2012; Kim et al., 2017). Raising at 450°C, the peak of CZTS was decreased and at the wavenumber of 192 cm^−1^ could be attributed to the CZTSSe phase. As the selenization temperature elevated, the vibration peak of CZTS disappeared, and the vibration signal intensity at 170 cm^−1^ and 192 cm^−1^ enhanced. It indicated that the entry of selenium into the lattice of CZTS increased the lattice constant and further caused the vibration peak of CZTS to move toward CZTSSe. In addition, the CZTSSe thin film annealed at 550°C showed sharper symmetrical vibration peaks around 170 cm^−1^ and 192 cm^−1^, and the vibration signal of CZTS disappeared near 336 cm^−1^. Corresponding to the CZTSSe phase, the transformation from CZTS to CZTSSe phase was achieved.

### Selenization Effect Over Different Selenization Times

Table 1 shows the atomic ratios of CZTSSe thin films at different selenization holding times. The stoichiometric ratio of Cu/(Zn + Sn) and Zn/Sn had no significant changes during the annealing process, indicating that all the selenization time was unable to cause the metal atoms of thin films to volatilize. In addition, the ratio of S/(S + Se) had a tendency to decrease under the prolongation of holding time, which demonstrated that the more the selenium doping is, the more easy the CZTSSe particle generating is. However, the forbidden bandwidth of CZTSSe decreases with the increase of selenium content in the thin films. According to theoretical research, when S/(S + Se) approaches 0.2, the efficiency of theory conversion can be increased (Pakštas et al., 2020). We achieved this condition through 60 min selenization holding time.

**TABLE 1:** Atomic ratios of CZTSSe thin films at different selenization holding times.

T (min)	Cu/(Zn + Sn)	Zn/Sn	S/(S + Se)	(S + Se)/metal
30	0.94	1.14	0.05	0.96
60	0.86	0.84	0.11	0.89
90	0.89	0.87	0.09	0.88
120	0.89	0.92	0.03	0.96

**FIGURE 5:**
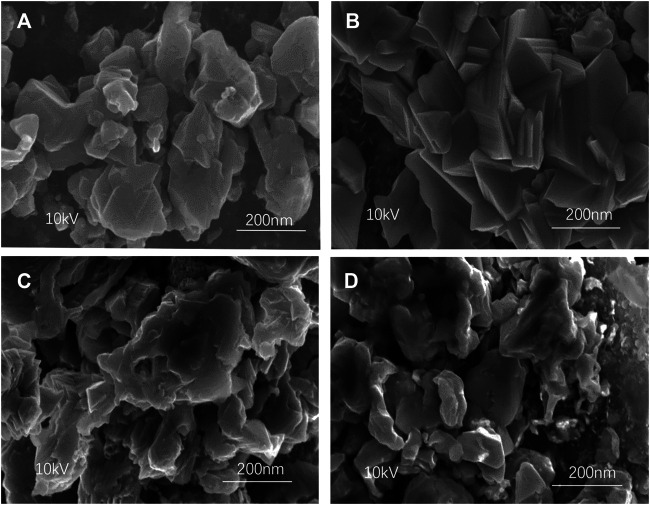
SEM images of CZTSSe thin films annealed at different times: **(A)** 30 min, **(B)** 60 min, **(C)** 90 min, and **(D)** 120 min.

Figure 5 shows the SEM images of CZTSSe thin films at different holding times. It can be seen from Figure 5A that the morphology of CZTSSe nanoparticles had been changed to tetragonal crystal at 30 min of selenization holding time. The sample formed a regular crystal when the selenization holding time reached 60 min, which certificated the fulfillment of the selenization process. As shown in Figures 5B–D, with all slight deformation, the CZTSSe maintained a better original morphology and crystallinity under 60 min of selenization holding time, which may be assigned to an appropriate time for selenium preservation. Moreover, it can be seen from Figure 5C that the surface of CZTSSe thin films became rough derived from the deposition of excessive metal selenide due to long insulation time. Therefore, with reference to the analysis of the SEM images, 60 min was the best selenization holding time.

**FIGURE 6:**
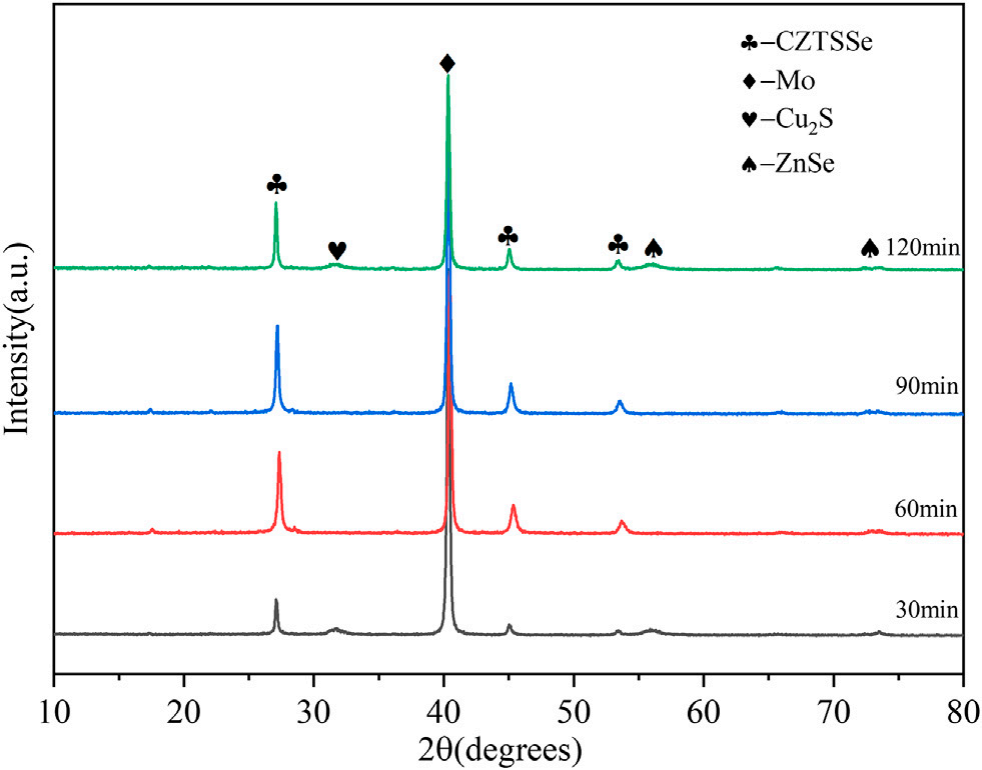
XRD patterns of CZTSSe thin films annealed at different times 30, 60, 90, and 120 min.

Figure 6 shows the XRD patterns of CZTSSe thin films at different selenization holding times. By comparing standard PDF cards (CZTS: JCPDS No.26–0575 and CZTSe: JCPDS No.52–0868), the three strong peaks in the figure correspond to the card position, and the formation of CZTSSe can be initially determined. However, the second phase of Cu_2_S and ZnSe was obvious to be found at the selenium holding time of 30 min, which proved that Se atoms were not replaced by S atoms but formed hetero phases such as Cu_2_S and ZnSe.

**FIGURE 7:**
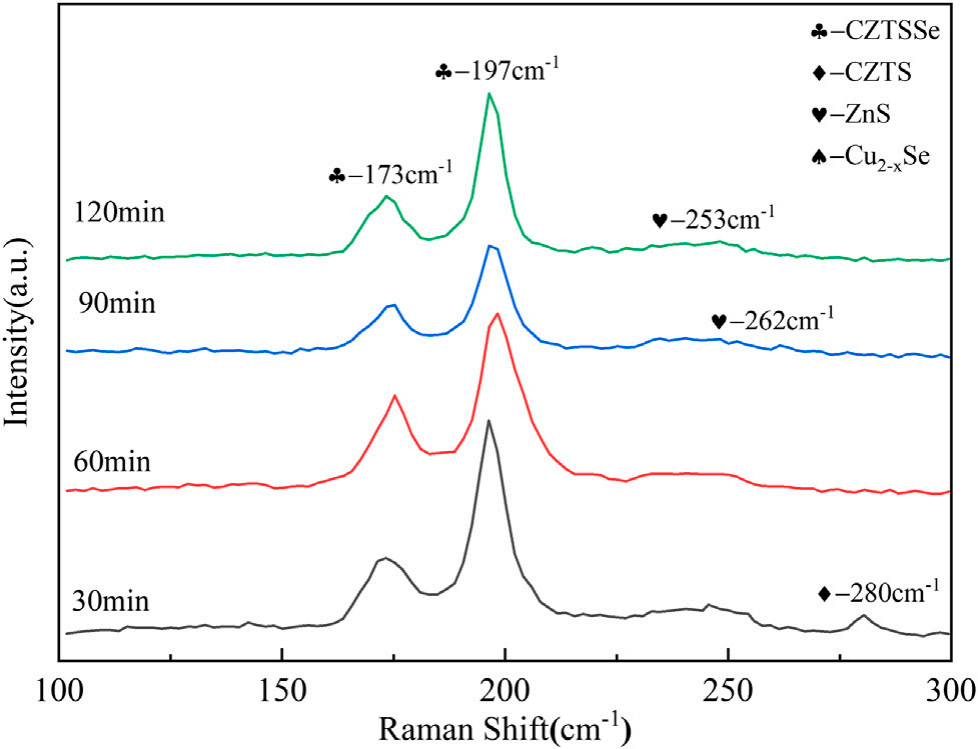
Raman spectra of CZTSSe thin films annealed at different times 30, 60, 90, and 120 min.

As can be seen from Figure 7, the vibration peak of 280 cm^−1^ did not disappear at 30 min, indicating the incomplete transformation from CZTS to CZTSSe; nevertheless, the vibrations of CZTSSe appeared at 173 cm^−1^ and 197 cm^−1^ (Kumar et al., 2018; Li et al., 2020). All of those proved the presence of residual CZTS; therefore, a large number of fine particles of CZTS still existed on the surface of the thin film. When the holding time reached above 60 min, the CZTS vibration peak disappeared; however, Cu_x_Se and ZnS peaks appeared annealed at 90 and 120 min (Chalapathy et al., 2018; Kogler et al., 2019). It is worth considering that more selenium particles may be evaporated with prolongation of the selenium holding time, resulting in the appearance of impurity peaks. Therefore, the optimal selenization holding time was 60 min.

### Selenization Effect Under Different Source Temperatures

As shown in Table 2, the content of Cu was minimal when the temperature of the selenium source was 400°C, while the ratio of S/(S + Se) tends to increase with rising selenium source temperature, and the ratio is close to 0.2, which was beneficial to the formation of CZTSSe thin films (Pakštas et al., 2020).

**TABLE 2:** Atomic ratios of CZTSSe thin films at different selenization source temperatures.

T (°C)	Cu/(Zn + Sn)	Zn/Sn	S/(S + Se)	(S + Se)/metal
300	0.97	1.24	0.16	0.91
350	0.96	0.84	0.08	1.04
400	0.86	0.85	0.11	0.89
450	0.95	0.82	0.14	1.11

**FIGURE 8:**
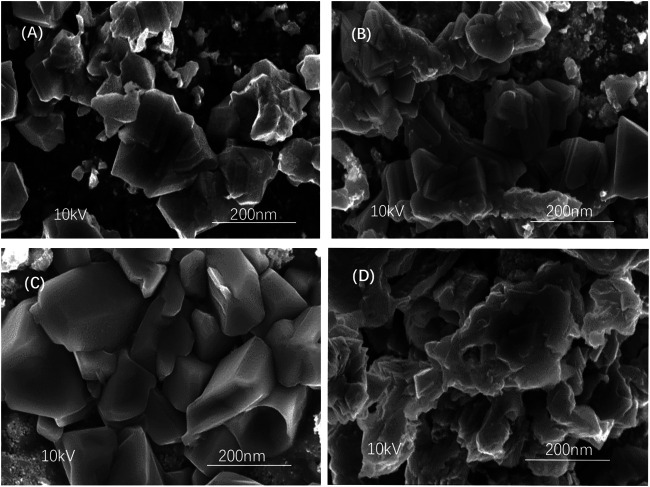
SEM images of CZTSSe thin films at different selenium source temperatures: **(A)** 300°C, **(B)** 350°C, **(C)** 400°C, and **(D)** 450°C.

SEM images in Figure 8 show the CZTSSe surface following different selenium source temperatures, 300, 350, 400, and 450°C. As shown in Figures 8A,B, samples with a selenium source temperature of 300 and 350°C exhibited some trivial particles, demonstrating the agglomeration with a nonuniform surface. Also, the sample at 450°C showed decreased crystallinity as shown in Figure 8D, resulting in deterioration of film morphology and surface roughness. When the selenium source temperature arrived at 400°C, the surface of the thin film was evenly to form a crystalline substance with an approximate size of 85 nm, which can inhibit carrier recombination and further improve the device efficiency of CZTSSe thin-film solar cells (Ren et al., 2020).

**FIGURE 9:**
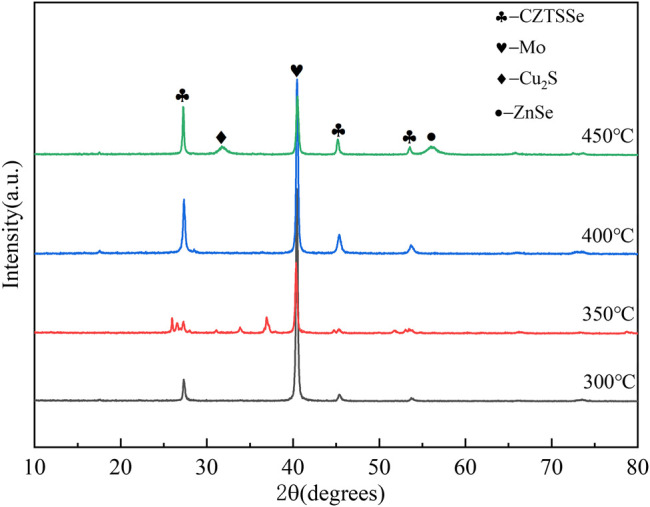
XRD patterns of CZTSSe thin films at different selenium source temperatures 300, 350, 400, and 450°C.

XRD confirmed that all samples annealed at different selenium source temperatures were highly crystalline as shown in Figure 9. The peak intensity of thin films increased with rising selenium source temperature due to promoting crystallinity of CZTSSe thin films. However, a secondary phase such as ZnSe occurred again at 450°C reducing the conversion efficiency of the CZTSSe thin-film solar cell.

**FIGURE 10:**
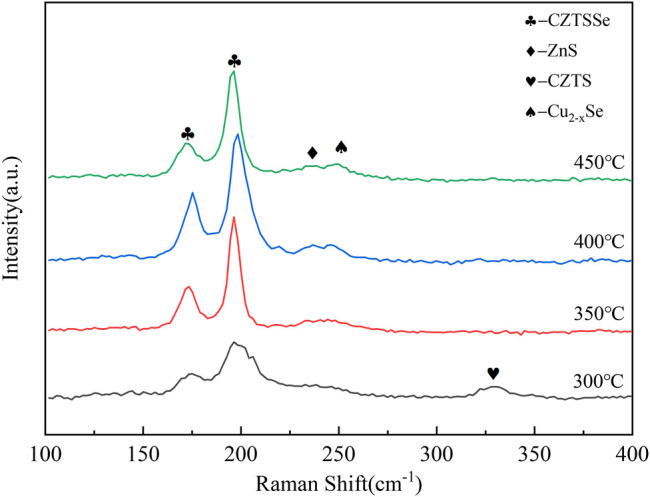
Raman spectra of CZTSSe thin films at different selenium source temperatures 300, 350, 400, and 450°C.

Figure 10 shows the Raman spectra for all samples. The Raman spectra of CZTSSe thin films under different selenium source temperatures showed obvious peaks at 170 cm^−1^ and 192 cm^−1^ associating with the main vibrational mode for single-phase CZTSSe. In addition, a weak peak appeared at 336 cm^−1^ at 300°C pointing to the residual of CZTS, and the weak of 336 cm^−1^ decreased following the increase of selenium source temperature. The above results indicated that Se doping into the CZTS lattice resulted in the migration of CZTS vibration peak to form CZTSSe. However, there were two distinct hetero peaks under the selenium source temperature at 450°C, certificating the formation of CuxSe and ZnS and further proving that excessive temperature of selenium source was not conducive to the composition of CZTSSe thin films. By studying the morphology, phase, and composition of CZTSSe thin films at different selenium source temperatures, it can be concluded that 400°C should be selected as the optimal temperature for the selenium source in the experiment.

**FIGURE 11:**
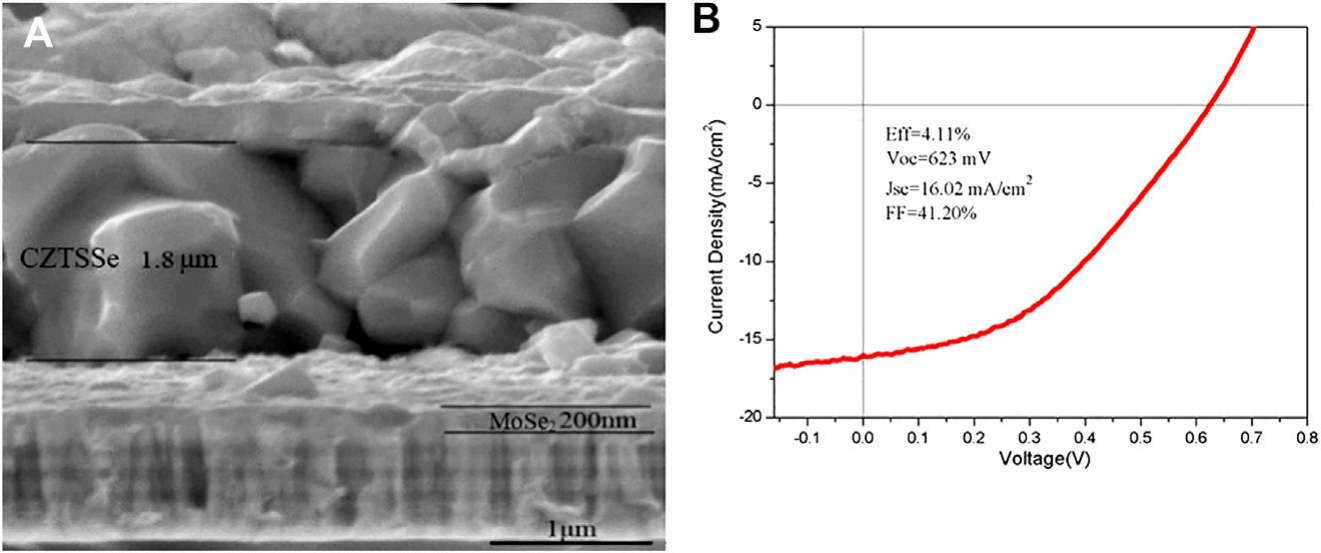
Cross section **(A)** and J-V curve **(B)** of CZTSSe thin-film solar cell.

Figure 11A shows the SEM cross section of the CZTSSe thin-film solar cell prepared by CZTO precursor and then annealed CZTS to form the Cu_2_ZnSn(S,Se)_4_(CZTSSe) thin film. The CZTSSe thin film maintained a thickness of 1.8 μm, which was suitable for the transformation of light energy. The CZTSSe thin film presented a large grain with tight contact of interfacial layers and no visible grain boundaries or pores. CZTSSe solar cell device was fabricated with a Mo/CZTSSe/CdS/i-ZnO/ITO/Ag, and the J-V curve was shown in Figure 11B. The device demonstrated 4.11% photoelectric conversion efficiency (PCE) with an open-circuit voltage (Voc) of 623 mV, a short circuit current density (Jsc) of 16.02 mA cm^−2^, and a fill factor (FF) of 41.2%. The low electron and hole bonding rate may be related to the large grain size, smooth surface, and dense thin-film morphology. In addition, ZnO can conduct holes, and the N-type CdS layer has the role of electron transfer, which further improves the electron transport efficiency. However, it is possible to form a second phase particle at the back contact interface by calcination of selenium such as MoSe_2_; the existence of a secondary phase has a negative impact on the performance of the solar cell and not only increases the resistance but also losses the quality of devices. Therefore, the CZTSSe thin film synthesized with the CZTO precursor under an optimal condition can ensure the condition of Cu-poor and Zn-rich, inhibit the formation of the second phase, reduce the self-compensation defect, and further improve the conversion efficiency of solar cells.

## Conclusion

In conclusion, the CZTO precursor can effectively promote the surface morphology of CZTSSe thin films. In the process of calcination, O atoms can be replaced by S atoms without any impurities creating high-quality CZTSSe thin films. It was revealed that the composition and morphology of the CZTO precursor played an important role in fabricating CZTSSe thin films with good crystallinity by controlling annealing temperature, holding time, and selenium source temperature. The CZTO precursor with suitable composition was obtained to regulate and control the formation of CZTSSe, which was confirmed by XRD, Raman, SEM, and EDX techniques. It was found that the peak of CZTSSe exists and becomes sharper with increasing calcination parameters (temperature, annealing time, and selenium source temperature). Raman curves proved the presence of CZTSSe thin films with two obvious peaks at 173 cm^−1^ and 197 cm^−1^; similar to the XRD test, the peak of CZTSSe also becomes stronger with the increase of annealing time. However, excessive or insufficient temperature and annealing times lead to the appearance of secondary phases such as ZnSn and Cu_x_Se. SEM scanning showed the phase transition of the thin film under different conditions and proved that large particle size and smooth and compact surface of the thin film can improve the performance of the CZTSSe solar cell. According to EDX analysis, with the change of annealing parameters, the composition of Cu/(Zn + Sn), Zn/Sn, and S/(S + Se) also changed to a varying degree. It can be found that the more the doped Se is, the more the CZTSSe is generated. Also, the forbidden bandwidth of the CZTSSe thin film decreases with the increase of Se atoms in the membrane; thus, the conversion efficiency of the CZTSSe thin-film solar cell was improved with the condition of S/(S + Se) approaching 0.2, the annealed temperature of 550°C at 60 min, and selenium source temperature of 400°C. Moreover, CZTSSe thin-film solar cells based on the periods of fabrication of CZTO precursor and selenization of the CZTS thin film demonstrated the power conversion efficiency of 4.11% due to complete diffusion of S and Se atoms. In conclusion, these confirm the feasibility of using CZTO precursor for the preparation of highly efficient CZTSSe solar cells by further optimal progression.

## Data Availability

The original contributions presented in the study are included in the article/Supplementary Material; further inquiries can be directed to the corresponding authors.
